# A real-time PCR method to genotype mutant mouse models with altered affinity for cardiotonic steroids on the Na,K-ATPase

**DOI:** 10.1371/journal.pone.0267348

**Published:** 2022-04-21

**Authors:** Peter W. Chomczynski, Kianna M. Vires, Michal Rymaszewski, Judith A. Heiny

**Affiliations:** 1 Molecular Research Center, Cincinnati, OH, United States of America; 2 Department of Pharmacology and Systems Physiology, University of Cincinnati, Cincinnati, OH, United States of America; Universidade Federal do Rio de Janeiro, BRAZIL

## Abstract

The highly conserved, cardiotonic steroid binding site (also termed ouabain binding site) on the primary α subunit of Na,K-ATPase plays a receptor signaling role in a range of vital cell processes and is a therapeutic target for human disease. Mouse lines with altered affinity for cardiotonic steroids on the α1 or α2 subunit isoform of Na,K-ATPase, without any change in pump activity, were developed by the late Jerry B Lingrel and are a valuable tool for studying its physiological roles and drug actions. In one model, the normally ouabain resistant α1 isoform was rendered sensitive to ouabain binding. In a second model, the normally sensitive α2 isoform was rendered resistant to ouabain binding. Additional useful models are obtained by mating these mice. To further advance their use, we developed a rapid, real-time PCR method that detects mutant alleles using specific primers and fluorescent probes. PCR is performed in fast mode with up to 15 samples processed in 40 min. The method was validated by Sanger sequencing using mice of known genotype, and by comparing results with a previous two-step method that used PCR amplification followed by gel electrophoresis. In addition, we clarified inconsistencies in published sequences, updated numbering to current reference sequences, and confirmed the continued presence of the mutations in the colony. It is expected that a wider availability of these models and a more efficient genotyping protocol will advance studies of the Na,K-ATPase and its cardiotonic steroid receptor.

## Introduction

The Na, K-ATPase (NKA) is an essential enzyme present in most cells of higher animals. It establishes the Na^+^ and K^+^ concentration gradients that underlie cell volume regulation, electrical signaling, and solute transport [1–3]. It also functions as a receptor for ouabain and related cardiotonic steroids (CTS) and this receptor is an important therapeutic target. High concentrations of ligands (millimolar range) inhibit pump transport, to produce both cytotoxic and therapeutic actions [4]; while concentrations far below saturation for inhibiting transport (≤ nM) activate a range of cell processes, including membrane trafficking, growth and proliferation, intracellular Ca^2+^ oscillations, cell signaling, and gene transcription [5–9]. These physiological roles of the CTS receptor remain incompletely understood and are the subject of active research. The Lingrel mouse models are an invaluable tool for such studies. They are especially useful because they enable studies of CTS receptor functions without a need to identify the endogenous ligand(s). Isolation and identification of endogenous CTS has proven challenging due to their extremely low circulating concentrations and the need for complex analytical separations [4].

The CTS receptor resides on the primary, ion-transporting α subunit of NKA. Four isoforms of the α subunit exist and show wide differences in affinity for CTS. In humans and many mammals, all α isoforms exhibit high affinity ouabain binding (apparent IC50 1–3 nM) [6]. However, in some rodents including mice, only α2 and α3 bind ouabain with high affinity, whereas α1 and α4 (sperm-specific) are resistant to ouabain binding (IC50 1000-fold greater) [10–12]. Both ouabain-sensitive and -insensitive isoforms conduct ion transport, and all α isoforms (α1-α4) are able to respond to nM ligand concentrations to elicit cell responses. [5–9, 13] The low affinity, resistant isoforms of rodents are protected from the transport- inhibiting, cytotoxic actions of higher CTS concentrations.

To advance studies of the physiological role(s) of the CTS receptor, the late Jerry B Lingrel and collaborators identified two critical amino acids in the receptor site that determine CTS binding [14] without any change in pump activity. Subsequently, they mutated α subunit genes to create knock-in mouse models in which the affinity of the CTS receptor for ouabain is altered, without any change in pump transport (**Table 1**) [15, 16]. Mutations in the mouse α1 gene (ATP1A1) convert its CTS receptor from low to high affinity ouabain binding (α1^S^, Sensitive); mutations in the mouse α2 gene (ATP1A2) convert it from high to low affinity ouabain binding (α2^R^, Resistant). Mating these mice produces additional useful models. These include the α1^S/S^α2^S/S^ “humanized” mouse with both isoforms sensitive, the α1^R/R^α2^R/R^ mouse with both isoforms resistant, the α1^S/S^α2^R/R^ “SWAP” mouse with reversed affinities [17], and their heterozygous (HET) combinations. These models have been used to uncover functional roles of the CTS receptor in the heart, vasculature, kidney, brain, and other cells and tissues [15, 18, 19]. They also enable studies of isoform-specific functions of NKA because they allow the researcher to selectively inhibit transport by either the α1 or α2 isoform using μM ouabain, to avoid cytotoxic effects of high ouabain concentrations [20]. The humanized model is useful for studies of CTS-derived drug candidates because both α1 and α2 isoforms are sensitive in humans. The SWAP model has been used to identify the NKA isoform that drives secondary transport processes such as Na^+^/Ca^2+^ exchange and Na-linked glucose transport [18, 19, 21, 22].

**Table 1:** Homozygous mouse models obtained by breeding mice with α1^S^ and/or α2^R^ mutations.

Genotype	α1^R/R^α2^S/S^	α1^R/R^ α2^R/R^	α1^S/S^α2^S/S^	α1^S/S^α2^R/R^
Phenotype	Resistant/Sensitive	Resistant/Resistant	Sensitive/Sensitive	Resistant/Sensitive
Description	WT	mutant α2, both isoforms resistant	mutant α1,both isoforms sensitive; “humanized”	double mutant, reversed affinities; “SWAP”
Original citations		[15]	[16]	[3]
Amino acid	none	**L116R** and **N127D**	**R118Q** and **D129N**	α1: **L116R** and **N127D**
substitution				α2: **R118Q** and **D129N**
(Prior notation)		(L111R and N122D)	(R111Q and D122N)	

Numerous additional HET combinations are possible. Numbering of amino acids is based on current reference sequences for murine NKA α1 (NP_659149.1) and α2 subunits (NP_848492.1) [23, 24]. The original amino acid numbering is shown in parentheses [15, 16].

Genotyping these many combinations using conventional methods is cumbersome and not always reliable. It is further complicated by inconsistencies in gene and primer sequences reported in the original literature, and outdated sequence numbering. The original method, consisting of PCR amplification followed by gel electrophoresis of the amplicons, does not directly detect the presence of the desired changes in the coding sequence; instead, it relies on observing the presence or absence of an upstream artificial insertion site (loxP site and padding) that correlates with the amino acid modifications. In addition, both **α1**^**S**^ and **α2**^**R**^ mutants, amplicons corresponding to the WT or mutant alleles differ in length by less than 50 bp, making it difficult at times to differentiate alleles by size on a gel.

Here, we developed a rapid and efficient genotyping protocol using a fluorescent probe- based, real-time PCR method. The method reliably differentiates offspring by direct detection of mutant alleles. Additionally, we performed Sanger sequencing to clarify these mutations and their associated genomic sequences and to confirm the continued presence of the mutations in the colony. It is expected that their wider availability and a more efficient method for genotyping will advance studies of the NKA and its CTS receptor.

## Materials and methods

The protocol described in this article is published on *protocols*.*io*, doi.org/10.17504/protocols.io.rm7vzym2rlx1/v3, and is included for printing as a S1 Appendix.

### Mice

Mice were generated as described [15, 16] and housed in pathogen-free conditions at the University of Cincinnati. All procedures involving animal were performed in accordance with the Guide for the Care and Use of Laboratory Animals (National Research Council of the National Academies, USA) and were approved by the Institutional Animal Care and Use Committee of the University of Cincinnati (IACUC Approval no. 07-05-07-08-01)

### Sequencing

DNA was obtained from mice of known status (n = 2 **α1**^**S**^, n = 2 **α2**^**R**^ mice and amplified by PCR using sequencing primers designed as described below. The resulting 4 amplicons were Sanger sequenced (MCLAB, San Francisco, CA) (forward and reverse direction at 4 loci). Results were analyzed and aligned with NCBI reference sequences and each-other.

### Genotyping

Primers and probes used for genotyping (**Table 2**) were designed using best practices [25]. They were designed to target the regions of each gene where the critical base- pair substitutions occur. Candidate sequences for primers and probes were chosen with PrimerQuest software and assessed for kinetic parameters (Tm, dimerization, hairpin loop formation) using OligoAnalyzer software [26]. Specificity was confirmed using NCBI BLAST [27]. Probes for α1 required locked nucleic acid (LNA) bases surrounding some SNP sites because a standard probe did not show sufficient specificity [28]. α1 probes were designed with assistance from IDT Application Support (Integrated DNA Technologies). All primers and probes were obtained from IDT. A melt-curve analysis using SYBR Green qPCR was performed to check each pair of primers for off-target amplification or excessive dimerization (Bio-Rad iTaq Universal SYBR Green Supermix, cat. no. 1725121). Each probe was then assessed for its ability to discriminate between wild-type and mutant alleles of its target gene. Candidate probes meeting these criteria were validated on multiple samples of known genotype. RT-PCR was performed using ABI StepOne and StepOnePlus real-time PCR machines (Thermo Fisher).

**Table 2:** Primers and probes.

Name	Amplicon Size and gene location	Sequence
ATP1A1 FWD primer	128 bp product: NC_000069.7[101499671‥101499798]	CAG CTC TTT GGA GGC TTT
ATP1A1 REV primer		GCT ACC GTA ACT ACA CAA CTC
ATP1A1 WT probe	α1^R^ allele probe	/56-FAM/CA+T +CC+G +A+AG T+GC /3IABkFQ/
ATP1A1 mutant probe	**α1**^**S**^ allele probe	/56-FAM/TGG AAT +TC+A +G+AG T+GC /3IABkFQ/
ATP1A2 FWD primer	103 bp product NC_000067.7[172118719‥172118821]	TCC TCT GCT TCT TAG CCT ATG G
ATP1A2 REV primer		CAG GGC TAT AAG CAG GTC CA
ATP1A2 WT probe	α2^S^ allele probe	/56-FAM/CAC ATT ATC /ZEN/GTT GGA TGG TTC GTC CTC C/3IABkFQ/
ATP1A2 mutant probe	**α2**^**R**^ allele probe	/56-FAM/CTC ACA TCA /ZEN/TCG TTC GAA GGC TCG TC/3IABkFQ/

“**+**” indicates a locked nucleic acid (LNA) before a base.“//”, indicates dye and quencher insertions; bp, base pair. Dye was 6-FAM, quenchers were ZEN and Iowa Black FQ. Locations are NCBI reference sequences accession numbers and positions.

## Results and discussion

**Fig 1:**
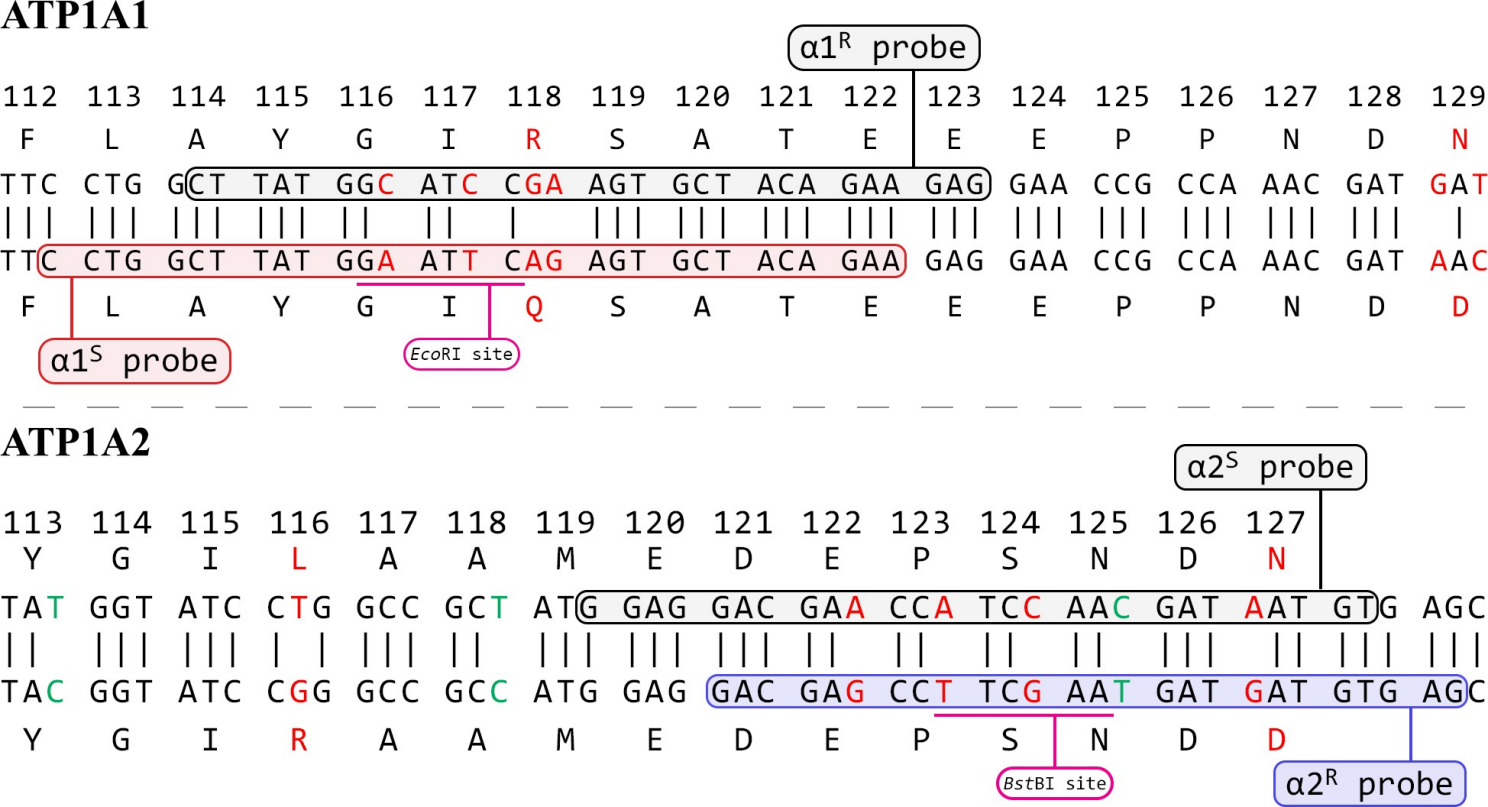
Sequence alignment of murine ATP1A and ATP1A2 variants and recognition sequences of the PCR probes. Amino acid numbering is based on current reference sequences for the murine NKA α1 and α2 subunits (from Table 1). ATP1A: gray highlight indicates the probe target site for the WT α1^R^ mutant; red highlight indicates the probe target site for the sensitive **α1**^**S**^ mutant. Base substitutions are indicated in red font. The presence or absence of vertical lines between sequences indicates whether a base change in the codon is conserved. ATP1A2: corresponding annotation. Blue highlight indicates the probe target site for the mutant **α2**^**R**^ mutant. Green font indicates natural variance between C57BL/6J and 129S1/SImJ mouse strains.

Sanger sequencing of the colony was performed to clarify and correct inconsistencies in initial reports [15, 16], to design PCR primers and probes, and to check the current status of critical mutations in the colony. **Fig 1** shows the sequence alignments for ATP1A (**α1**^**S**^) and ATP1A2 (**α2**^**R**^) mutants and the target hybridization sequence of each newly designed probe. Results confirmed that the critical SNPs remain in the colony.

The WT α1^R^ allele is identical to the NCBI reference sequence at this locus for the C57BL/6J strain. The mutant **α1**^**S**^ allele differs from WT at 6 base pairs; 2 changes were introduced to alter the protein affinity and 4 to insert a restriction enzyme site. The WT α2^S^ allele is identical to the corresponding reference sequence for the C57BL/6J strain. However, the **α2**^**R**^ allele derives from the 129S1/SvImJ strain; it includes 3 silent mutations (highlighted in green) not seen in C57BL/6J. The allele contains 5 artificially introduced base-pair changes that alter the protein affinity and create a restriction enzyme site. Several individuals were sequence, and none had a WT allele from the 129S1/SvImJ strain. Sequencing of a region upstream of the α2 gene confirmed this identification.

**Fig 2:**
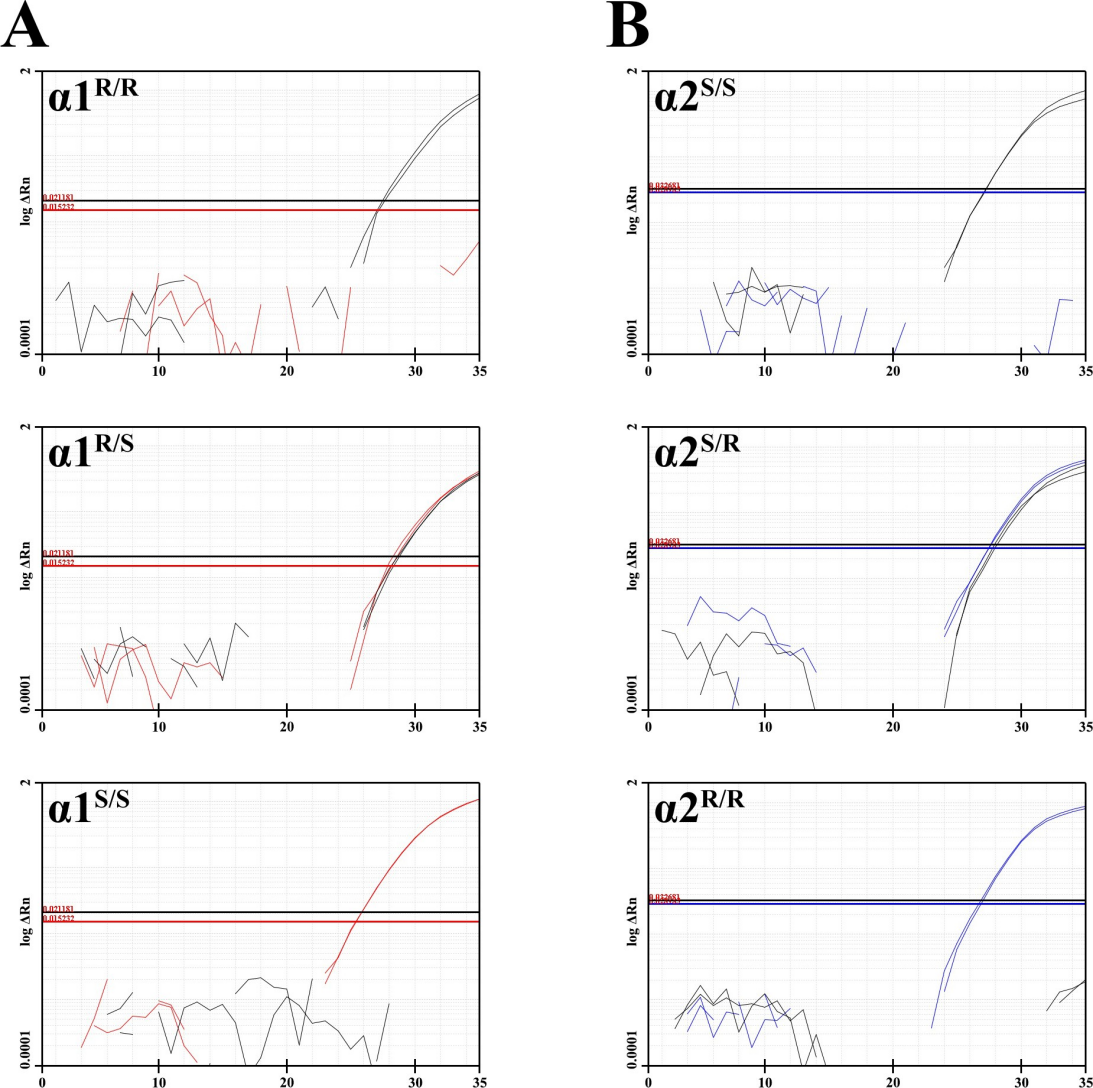
Detection of α1^S^ and α2^R^ mutants by qPCR using mutant-specific fluorescent probes. ΔRn, fluorescence level vs. cycle number (C_T_). Horizontal lines indicate threshold fluorescence level for the WT (black), **α1**^**S**^ (red) and **α2**^**R**^ (blue) probes. Samples considered positive for a specific allele showed C_T_ in the range of 26-31for the corresponding probe, while negative samples did not reach threshold in 35 cycles; heterozygous samples had amplification of both probes within 2 C_T_ of each-other. The curves from both probes (run separately, in duplicate) of each gene are superimposed onto one plot. **A)** Amplification plots from the α1 (ATP1A1) assay of 3 samples representative of WT, HET, and homozygous mutant genotypes; α1^R^ probe (black), **α1**^**S**^ probe (red). **B)** Amplification plots from the α2 (ATP1A2) assay of 3 samples representative of WT, HET, and homozygous mutant genotypes; α2^S^ probe (black), **α2**^**R**^ probe **(**blue).

**Fig 2** illustrates the method for genotyping the **α1**^**S**^ and **α2**^**R**^ mutants by real-time fast PCR. A sample obtained from tail clips is digested and subjected to PCR without DNA isolation. PCR amplification is performed in fast mode using mutant-specific primers and probes (Table 2). Presence or absence of fluorescence indicates probe hybridization to the target, as measured by cycle number (C_T_).

The method is fast and efficient. It can genotype 11 samples for both genes, in duplicate, using a 96-well plate (including negative controls) in 40 min cycling time. To save reagents, it is possible to multiplex the WT α1^R^ and α2^S^ probes in one reaction if the α1^R^ probe is ordered with SUN dye (a molecular equivalent to VIC). This requires both sets of primers to be included in its reaction mix. Multiplexing allows for up to 15 samples (including negative controls) per 96-well plate.

To validate the method, we analyzed samples having known genotypes using the new real-time PCR method and compared the results with those of the previous two-step gel electrophoresis method. Results showed 100% agreement (n = 27 mice with α1 genotypes, n = 19 mice with α2 genotypes; 3 replicates analyzed per mouse) and agreed with the sequences obtained by Sanger sequencing.

## Conclusions

This report introduces a fast and efficient method for genotyping mouse models with altered affinity for cardiotonic steroids on the NKA **α1**^**S**^ and **α2**^**R**^ subunit isoforms. The method uses PCR amplification of digested tail samples with fluorescent probe-based detection of the critical mutations. The method, validated by sequencing, provides a substantially simplified and accurate protocol for genotyping the models. It also clarifies and corrects inconsistencies in the gene, amino acid, and primer sequences previously reported.

## Supporting information

S1 Appendix (PDF)
