# Supplementary material for: A real-time PCR method to genotype mutant mouse models with altered affinity for cardiotonic steroids on the Na,K-ATPase
Source: PLoS One. 2022 Apr 21;17(4):e0267348. doi: 10.1371/journal.pone.0267348 (PMC9022855; doi:10.1371/journal.pone.0267348)
Supplement: S1 Appendix — (PDF) [file pone.0267348.s001.pdf]

A real-time PCR method to genotype mutant mouse models with altered affinity for cardiotonic steroids on the Na,K-ATPase V.3

3

Apr 02, 2022

Peter W W Chomczynski[1], Kianna M Vires[2], Michal Rymascewski[3], Judith A. Heiny[2]

[1]Molecular Research Center, inc.; [2]University of Cincinnati; [3]Molecular Research Center, Inc.

1

dx.doi.org/10.17504/protocols.io.rm7vzym2rlx1/v3

Molecular Research Center, inc.

Peter W Chomczynski

The highly conserved, cardiotonic steroid binding site (also termed ouabain binding site) on the primary α subunit of Na,K-ATPase plays a receptor signaling role in a range of vital cell processes and is a therapeutic target for human disease. Mouse lines with altered affinity for cardiotonic steroids on the α1 or α2 subunit isoform of Na,K-ATPase, without any change in pump activity, were developed by the late Jerry B Lingrel and are a valuable tool for studying its physiological roles and drug actions. In one model, the normally ouabain resistant α1 isoform was rendered sensitive to ouabain binding. In a second model, the normally sensitive α2 isoform was rendered resistant to ouabain binding. Additional useful models are obtained by mating these mice. To further advance their use, we developed a rapid, real-time PCR method that detects mutant alleles using specific primers and fluorescent probes. PCR is performed in fast mode with up to 15 samples processed in 40 min. The method was validated by Sanger sequencing using mice of known genotype, and by comparing results with a previous two-step method that used PCR amplification followed by gel electrophoresis. In addition, we clarified inconsistencies in published sequences, updated numbering to current reference sequences, and confirmed the continued presence of the mutations in the colony. It is expected that a wider availability of these models and a more efficient genotyping protocol will advance studies of the Na,K-ATPase and its cardiotonic steroid receptor.

DOI

dx.doi.org/10.17504/protocols.io.rm7vzym2rlx1/v3

Citation: Peter W W Chomczynski, Kianna M Vires, Michal Rymascewski, Judith A. Heiny A real-time PCR method to genotype mutant mouse models with altered affinity for cardiotonic steroids on the Na,K-ATPase https://dx.doi.org/10.17504/protocols.io.rm7vzym2rlx1/v3

This is an open access protocol distributed under the terms of the **Creative Commons Attribution License**

Peter W W Chomczynski, Kianna M Vires, Michal Rymascewski, Judith A. Heiny 2022. A real-time PCR method to genotype mutant mouse models with altered affinity for cardiotonic steroids on the Na,K-ATPase. **protocols.io**
https://dx.doi.org/10.17504/protocols.io.rm7vzym2rlx1/v3
Peter W Chomczynski

SWAP mouse, ATP1A1, ATP1A2, Ouabain binding site, Mouse models, NaK ATPase

protocol ,

Diagrams created by BioRender.com. Screenshots from ABI StepOne software.

Apr 02, 2022

Apr 02, 2022

60231

Common reagents:
1M Tris pH 8.0
0.5 M EDTA pH 8.0
5 M NaCL
SDS 10% w/v
NP40 detergent (10%)
Tween-20
proteinase K (20 mg/mL)
ddH2O
Bio-Rad iTaq Universal Probes Supermix (cat. no. 1725131) or compatible equivalent

Protocol-specific primers and probes:
ATP1A1 FWD primer (100 mM): CAG CTC TTT GGA GGC TTT
ATP1A1 REV primer (100 mM): GCT ACC GTA ACT ACA CAA CTC
ATP1A1 WT probe (100 mM): /56-FAM/CA+T +CC+G +A+AG T+GC /3IABkFQ/
ATP1A1 mutant probe (100 mM): /56-FAM/TGG AAT +TC+A +G+AG T+GC /3IABkFQ/
ATP1A2 FWD primer (100 mM): TCC TCT GCT TCT TAG CCT ATG G
ATP1A2 REV primer (100 mM): CAG GGC TAT AAG CAG GTC CA
ATP1A2 WT probe (100 mM): /56-FAM/CAC ATT ATC /ZEN/GTT GGA TGG TTC GTC CTC C/3IABkFQ/
ATP1A2 mutant probe (100 mM): /56-FAM/CTC ACA TCA /ZEN/TCG TTC GAA GGC TCG TC/3IABkFQ/

Pre-experiment preparation

Citation: Peter W W Chomczynski, Kianna M Vires, Michal Rymascewski, Judith A. Heiny A real-time PCR method to genotype mutant mouse models with altered affinity for cardiotonic steroids on the Na,K-ATPase
https://dx.doi.org/10.17504/protocols.io.rm7vzym2rlx1/v3

This is an open access protocol distributed under the terms of the **Creative Commons Attribution License**

1 In the interest of time and consistency, it is recommended that certain stock solutions and buffers be prepared ahead.

1.1 Prepare a **10X stock solution of Tail Lysis Buffer** and store in freezer. 15m

Reagent	Vol. to make 10 mL
1M Tris pH 8.0	1 mL
0.5 M EDTA pH 8.0	2 mL
5 M NaCL	2 mL
SDS 10% w/v	5 mL

Tail Lysis Buffer, 10X stock solution

1.2 20m

Prepare **assay mixes**.
Primers and probes should be pre-mixed and stored frozen for convenience. Be mindful of the reagent stock concentrations to ensure a successful outcome. Mixes can be scaled as necessary.

Reagent	For 200 rxn
ATP1A1 FWD primer (100 µM)	20 µL
ATP1A1 REV primer (100 µM)	20 µL
ATP1A1 WT probe (100 µM)	10 µL
ddH_2O	150 µL

$\alpha 1^R$ assay mix

Citation: Peter W W Chomczynski, Kianna M Vires, Michal Rymascewski, Judith A. Heiny A real-time PCR method to genotype mutant mouse models with altered affinity for cardiotonic steroids on the Na,K-ATPase https://dx.doi.org/10.17504/protocols.io.rm7vzym2rlx1/v3

This is an open access protocol distributed under the terms of the **Creative Commons Attribution License**

Reagent	For 200 rxn
ATP1A1 FWD primer (100 µM)	20 µL
ATP1A1 REV primer (100 µM)	20 µL
ATP1A1 mutant probe (100 µM)	10 µL
ddH_2O	150 µL

α1^S assay mix

Reagent	For 200 rxn
ATP1A2 FWD primer (100 µM)	20 µL
ATP1A2 REV primer (100 µM)	20 µL
ATP1A2 WT probe (100 µM)	10 µL
ddH_2O	150 µL

α2^S assay mix

Reagent	For 200 rxn
ATP1A2 FWD primer (100 µM)	20 µL
ATP1A2 REV primer (100 µM)	20 µL
ATP1A2 mutant probe (100 µM)	10 µL
ddH_2O	150 µL

α2^R assay mix

1.3 Prepare **Tail Digestion Buffer** fresh for each experiment, per the following table. Add proteinase K last from a frozen and thawed aliquot. 15m

Citation: Peter W W Chomczynski, Kianna M Vires, Michal Rymascewski, Judith A. Heiny A real-time PCR method to genotype mutant mouse models with altered affinity for cardiotonic steroids on the Na,K-ATPase
https://dx.doi.org/10.17504/protocols.io.rm7vzym2rlx1/v3

This is an open access protocol distributed under the terms of the **Creative Commons Attribution License**

Reagent	Vol. to make 10 mL
10X Tail Lysis Buffer *	1 mL
NP40	45 µL
Tween-20	45 µL
proteinase K (20 mg/mL)	200 µL
ddH2O	to 10 mL

Tail Digestion Buffer
* final working concentrations are: 10 mM Tris pH 8, 10 mM EDTA, 100 mM NaCl, 0.5% SD

Tail clip digestion 12h 45m

2 Clip 2-3 mm from the tail of each mouse to be genotyped. Place in a clean, labelled 1.5 mL microcentrifuge tube. 30m

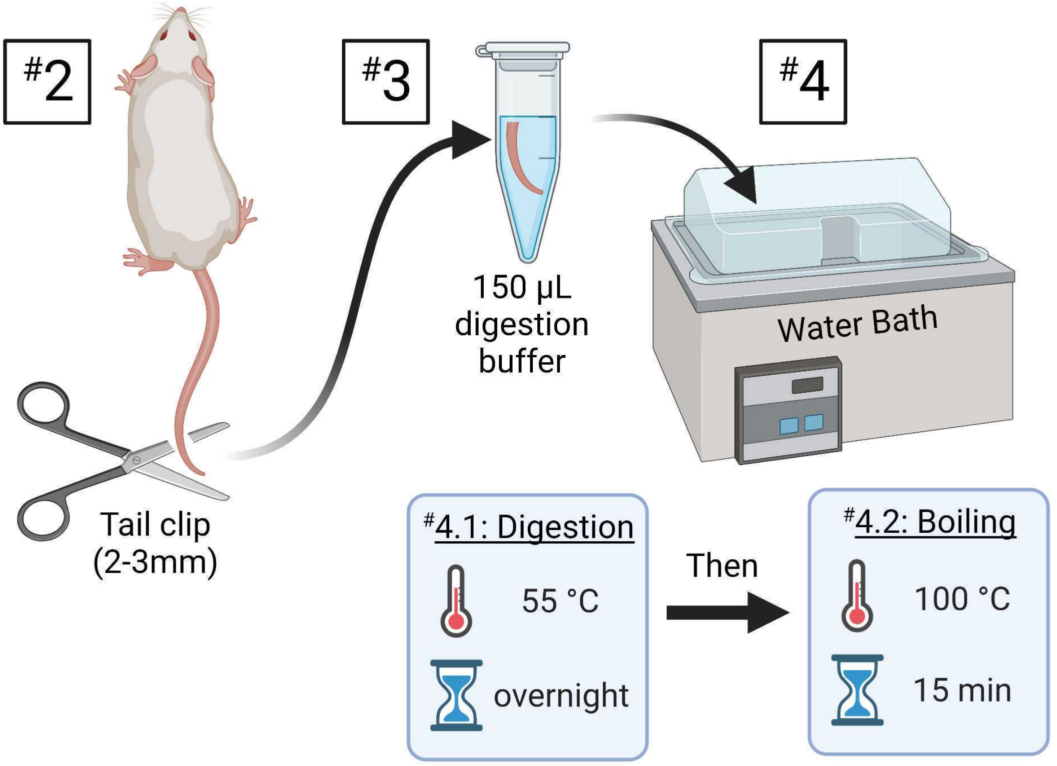

Steps 2-4 visual overview

3 Add **150 µL** of **Tail Digestion Buffer** containing proteinase K to each tube. 5m

Citation: Peter W W Chomczynski, Kianna M Vires, Michal Rymascewski, Judith A. Heiny A real-time PCR method to genotype mutant mouse models with altered affinity for cardiotonic steroids on the Na,K-ATPase
https://dx.doi.org/10.17504/protocols.io.rm7vzym2rlx1/v3

This is an open access protocol distributed under the terms of the **Creative Commons Attribution License**

4 Place the tail samples in a heat block or water bath. 3m

4.1 Incubate Overnight at 55 °C . 15m

4.2 Raise the temperature to 100 °C for 00:15:00 . 15m

4.3 Remove the tubes and allow to cool to Room temperature . 10m

5 Briefly vortex and centrifuge the tubes to pellet insoluble material. DNA will remain in the supernatant. 5m

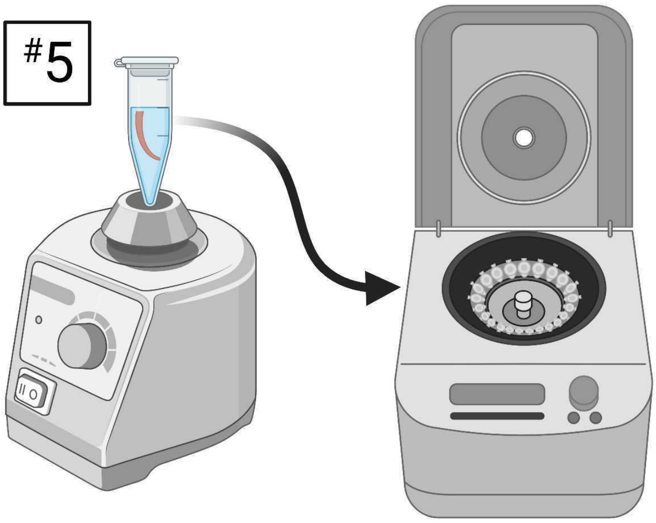

6 Create a 1:100 dilution of each sample in a new, labelled 1.5 mL microcentrifuge tube. Add 198 µL of TE buffer (or ddH2O) to each tube, then add 2.0 µL of supernatant from the digested sample. 10m

Citation: Peter W W Chomczynski, Kianna M Vires, Michal Rymascewski, Judith A. Heiny A real-time PCR method to genotype mutant mouse models with altered affinity for cardiotonic steroids on the Na,K-ATPase
https://dx.doi.org/10.17504/protocols.io.rm7vzym2rlx1/v3

This is an open access protocol distributed under the terms of the **Creative Commons Attribution License**

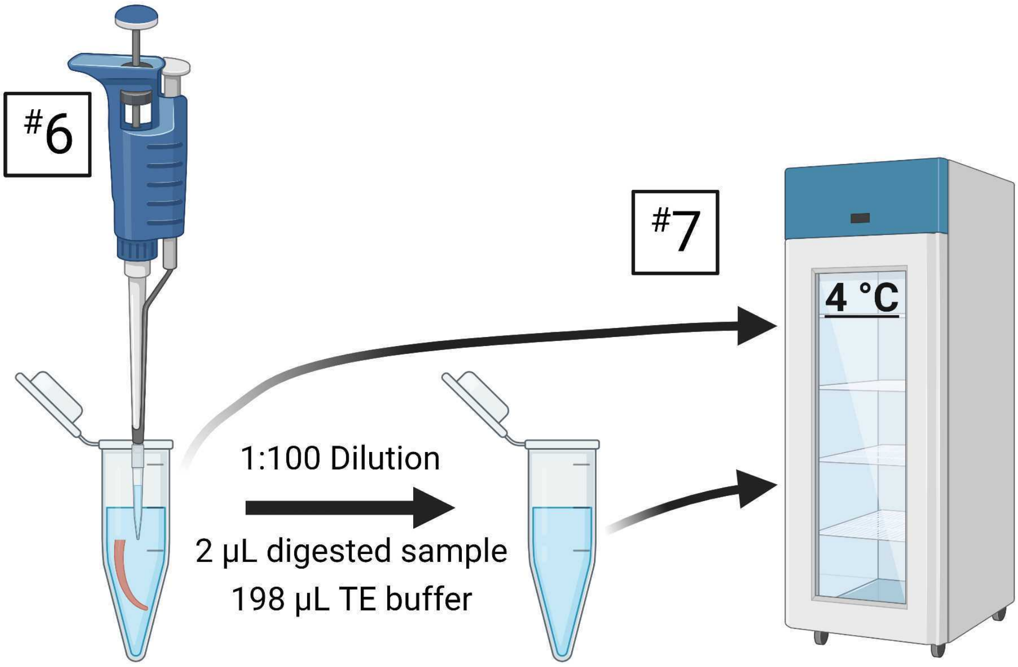

7 Store samples and dilutions at 4 °C until ready to genotype. If storing samples longer than 14 days, keep in -20 °C freezer.

PCR machine setup

8 Create a new experiment on your PCR machine's software. The instructions here are based on ABI StepOne machines but other manufacturers' procedures are generally similar. 2m

Templates for ABI StepOne and StepOnePlus machines are attached, which have the probe list and thermocycling program pre-set. To use the templates, download the version corresponding to your machine type. Open StepOne Software and choose [File] -> [New Experiment] -> [From Template...] and select the template file.

SWAP mouse genotype StepOnePlus template.edt

SWAP mouse genotype StepOne template.edt

9 Set the thermocycling program (run method) as per the following table. 2m

Citation: Peter W W Chomczynski, Kianna M Vires, Michal Rymascewski, Judith A. Heiny A real-time PCR method to genotype mutant mouse models with altered affinity for cardiotonic steroids on the Na,K-ATPase https://dx.doi.org/10.17504/protocols.io.rm7vzym2rlx1/v3

This is an open access protocol distributed under the terms of the **Creative Commons Attribution License**

Stage	Duration (m:ss)	Temperature	Cycles
Initial denaturation	3:00	95 C	1
Denaturation	0:03	95 C	35 Cycles
Annealing/Extension	0:30	95 C	

10 Enter the 4 probes into the targets list as per the following table. Populate the sample list with your mouse sample numbers, plus a no-template control (NTC) sample. 2m

Probe	Reporter	Quencher
a1R	VIC	None
a1S	FAM	None
a2S	FAM	None
a2R	FAM	None

Genotyping probe configurations

11 Assign 8 wells for each sample (including the NTC); 2 wells for $a1^R$, 2 wells for $a1^S$, 2 wells for $a2^S$, and 2 wells for $a2^R$. For the NTC, mark the wells as negative controls for the respective targets. 5m

A sample plate layout for genotyping 5 mice is shown here (ABI StepOne).

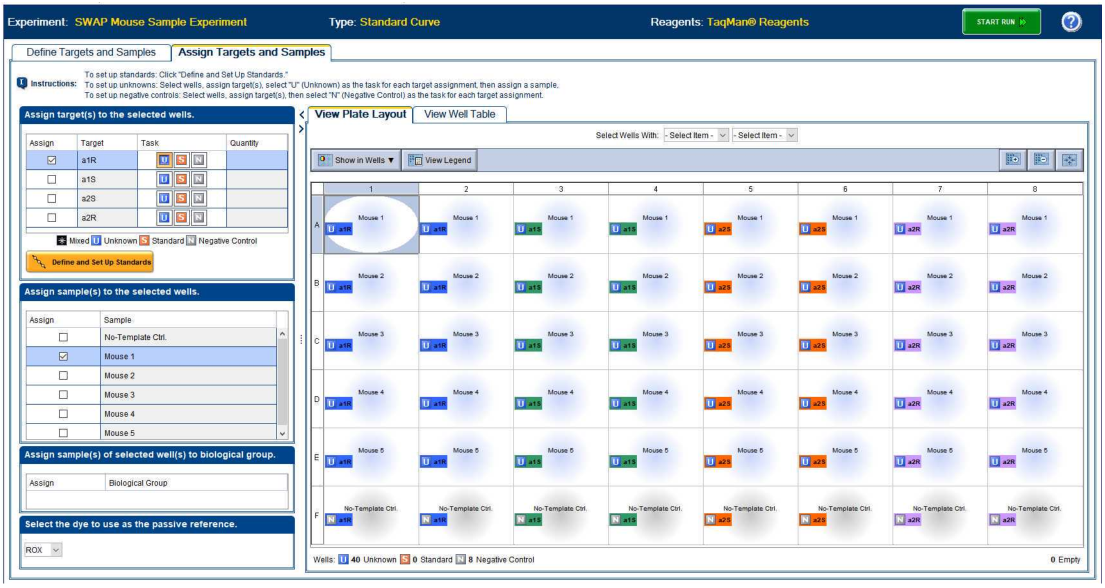

Citation: Peter W W Chomczynski, Kianna M Vires, Michal Rymascewski, Judith A. Heiny A real-time PCR method to genotype mutant mouse models with altered affinity for cardiotonic steroids on the Na,K-ATPase
https://dx.doi.org/10.17504/protocols.io.rm7vzym2rlx1/v3

This is an open access protocol distributed under the terms of the **Creative Commons Attribution License**

PCR reaction setup 2m

12 Prepare 4 reaction mixes as follows, 1 for each of the assay mixes. Create enough mix for the total number of reactions of each assay on your plate map, plus 2 extra to account for potential pipetting loss. 15m
For example, the plate shown in Step 11 (having 5 samples + 1 NTC) would need a 14 rxn mix for each assay. A full 96-well plate with 11 mouse samples and 1 NTC would require a 26 rxn mix for each assay.

Reagent	1 rxn	14 rxn	26 rxn
iTaq Probe mix (2X)	10.0 µL	140 µL	260 µL
Assay mix	1.0 µL	14 µL	26 µL
ddH2O	4.0 µL	56 µL	104 µL

PCR reaction mix

13 Pipet **15 µL** reaction mix into each well of a fresh PCR plate. 5m

14 Pipet **5 µL** of each diluted sample into its assigned wells. Pipet **5 µL** ddH2O into each no-template control well. 7m

15 Cover the plate with optical sealing film. Ensure that the film is fully adhered to the plate. 1m

16 Briefly vortex the plate. 1m

17 Centrifuge the plate at **1700 rpm, 00:02:00** . 2m

18 Insert the plate into the instrument and initiate the run. 1h 45m

Citation: Peter W W Chomczynski, Kianna M Vires, Michal Rymascewski, Judith A. Heiny A real-time PCR method to genotype mutant mouse models with altered affinity for cardiotonic steroids on the Na,K-ATPase https://dx.doi.org/10.17504/protocols.io.rm7vzym2rlx1/v3

This is an open access protocol distributed under the terms of the **Creative Commons Attribution License**

Interpretation

19 Analysis of the PCR results begins by checking the outcome of the negative control wells. If all NTC wells show no amplification, you may proceed with the analysis. If one or more negative control wells show amplification, the results are invalid and the experiment must be repeated. An invalid outcome is typically due to contamination of the reaction mix or of a reagent.

20 Genotyping is based on the presence or absence of signal from the 2 probes of each gene. The example below shows representative amplification plots for the possible genotypes of α1 (**A**) and α2 (**B**). The graphs show results from a sample's α1^{R} (black) and α1^{S} (red) wells plotted together (**A**), and a sample's α2^{S} (black) and α2^{R} (blue) wells plotted together (**B**).

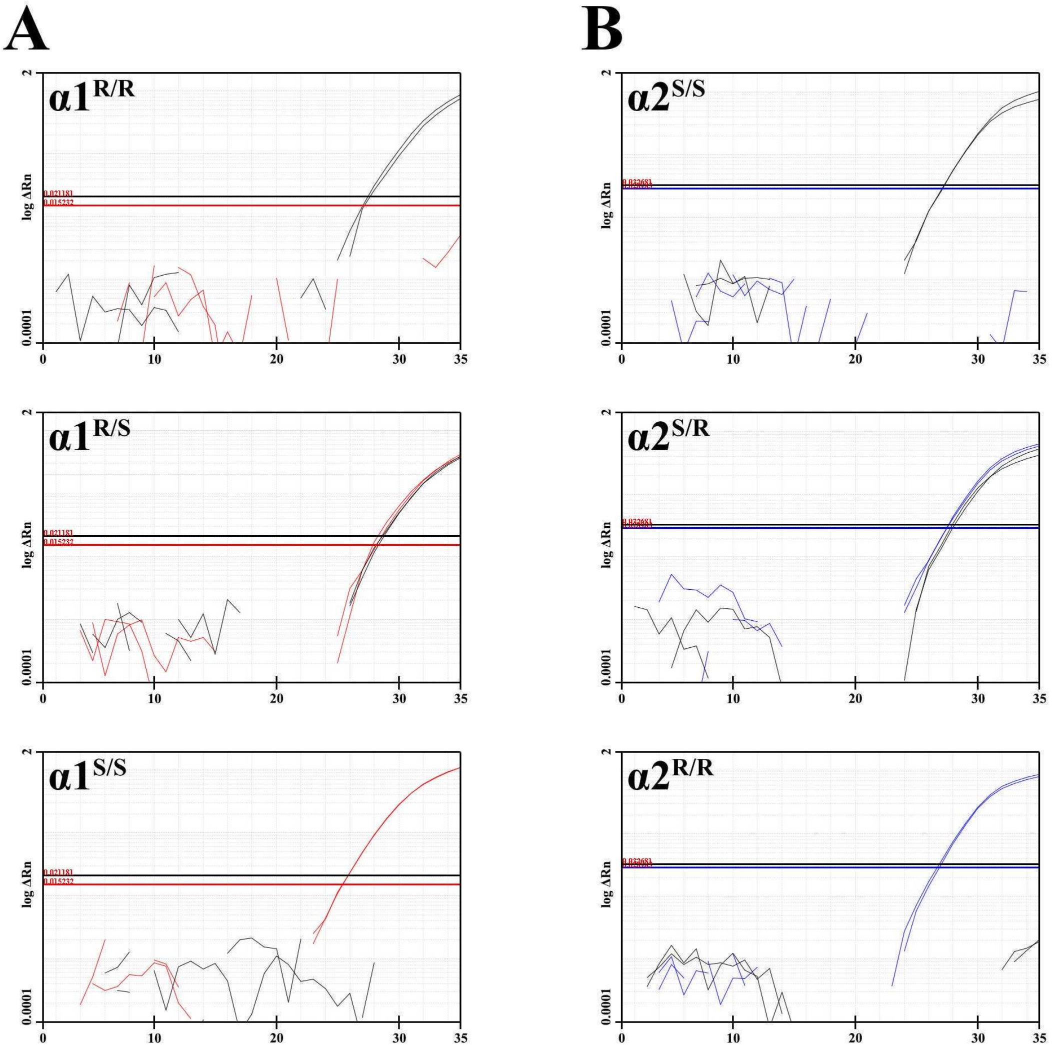

Representative amplification curves

Samples considered positive for a specific allele show C_T values of 26-31 for the corresponding probe, while negative samples do not reach threshold in 35 cycles. Heterozygous samples show amplification of both probes within 2 C_T of each-other.

Citation: Peter W W Chomczynski, Kianna M Vires, Michal Rymascewski, Judith A. Heiny A real-time PCR method to genotype mutant mouse models with altered affinity for cardiotonic steroids on the Na,K-ATPase https://dx.doi.org/10.17504/protocols.io.rm7vzym2rlx1/v3

This is an open access protocol distributed under the terms of the **Creative Commons Attribution License**

Citation: Peter W W Chomczynski, Kianna M Vires, Michal Rymascewski, Judith A. Heiny A real-time PCR method to genotype mutant mouse models with altered affinity for cardiotonic steroids on the Na,K-ATPase
https://dx.doi.org/10.17504/protocols.io.rm7vzym2rlx1/v3

This is an open access protocol distributed under the terms of the **Creative Commons Attribution License**
